# Quality of life and quality-adjusted survival (Q-TWiST) in patients receiving dose-intensive or standard dose chemotherapy for high-risk primary breast cancer

**DOI:** 10.1038/sj.bjc.6604092

**Published:** 2007-11-27

**Authors:** J Bernhard, D Zahrieh, J J Zhang, G Martinelli, R Basser, C Hürny, J F Forbes, S Aebi, W Yeo, B Thürlimann, M D Green, M Colleoni, R D Gelber, M Castiglione-Gertsch, K N Price, A Goldhirsch, A S Coates

**Affiliations:** 1 IBCSG Coordinating Center, Effingerstrasse 40, Bern 3008, Switzerland; 2 Department of Medical Oncology, Inselspital, Bern, Switzerland; 3 Department of Biostatistics and Computational Biology, IBCSG Statistical Center, Dana-Farber Cancer Institute, 44 Binney Street, LW2, Boston, MA 02115, USA; 4 Department of Biostatistics, Harvard School of Public Health, 655 Huntington Avenue, SPH2, 4th Floor, Boston, MA 02115, USA; 5 Haematology Division, Department of Medicine, European Institute of Oncology, Milan I-20141, Italy; 6 Department of Medical Oncology, The Royal Melbourne Hospital and CSL, Victoria, Australia; 7 Bürgerspital, Rorschacherstrasse 94, 9000 St., Gallen, Switzerland; 8 Department of Surgical Oncology, Newcastle NSW 2310, Australia Australian New Zealand Breast Cancer Trials Group, University of Newcastle, Newcastle Mater Hospital, Locked Bag 7, Hunter Region Mail Centre, Newcastle, New South Wales, Australia; 9 Department of Medical Oncology, Inselspital, Bern 3010, Switzerland and Swiss Group for Clinical Cancer Research (SAKK); 10 Department of Clinical Oncology, Chinese University of Hong Kong, Prince of Wales Hospital, 30-32 Ngan Shing Street, Shatin New Territories, Hong Kong SAR, China; 11 Senology Center of Eastern Switzerland, Kantonsspital, 9007 St Gallen, Switzerland and Swiss Group for Clinical Cancer Research (SAKK); 12 Department of Medical Oncology, Private Medical Centre, Suite 32, Grattan Street The Royal Melbourne and Western Hospitals, Parkville Victoria 3052, Australia; 13 Department of Medicine, European Institute of Oncology, Milan I-20141, Italy; 14 Department of Biostatistics, Harvard School of Public Health, Frontier Science Technology and Research Foundation, Boston, MA, USA; 15 Frontier Science Technology and Research Foundation, Boston, MA, USA; 16 European Institute of Oncology, Milan, Italy; 17 Oncology Institute of Southern Switzerland, c/o Ospedale Italiano, Via Capelli, 6962 Viganello-Lugano, Bellinzona, Switzerland; 18 International Breast Cancer Study Group, Bern, Switzerland; 19 School of Public Health, University of Sydney, 40 Cook Road, Centennial Park NSW 2021, Australia

**Keywords:** breast cancer, adjuvant chemotherapy, quality of life, adaptation, quality-adjusted survival

## Abstract

Quality of life (QL) is an important consideration when comparing adjuvant therapies for early breast cancer, especially if they differ substantially in toxicity. We evaluated QL and Q-TWiST among patients randomised to adjuvant dose-intensive epirubicin and cyclophosphamide administered with filgrastim and progenitor cell support (DI-EC) or standard-dose anthracycline-based chemotherapy (SD-CT). We estimated the duration of chemotherapy toxicity (TOX), time without disease symptoms and toxicity (TWiST), and time following relapse (REL). Patients scored QL indicators. Mean durations for the three transition times were weighted with patient reported utilities to obtain mean Q-TWiST. Patients receiving DI-EC reported worse QL during TOX, especially treatment burden (month 3: *P*<0.01), but a faster recovery 3 months following chemotherapy than patients receiving SD-CT, for example, less coping effort (*P*<0.01). Average Q-TWiST was 1.8 months longer for patients receiving DI-EC (95% CI, −2.5 to 6.1). Q-TWiST favoured DI-EC for most values of utilities attached to TOX and REL. Despite greater initial toxicity, quality-adjusted survival was similar or better with dose-intensive treatment as compared to standard treatment. Thus, QL considerations should not be prohibitive if future intensive therapies show superior efficacy.

Patient-reported quality of life (QL) during adjuvant chemotherapy for early breast cancer shows transient adverse effects during therapy, but it improves after cessation of standard therapy (Hurny *et al*, 1996; Fairclough *et al*, 1999; de Haes *et al*, 2003; Bernhard *et al*, 2004, 2007; Land *et al*, 2004; Martin *et al*, 2005), dose-intensive therapy (Del Mastro *et al*, 2002), or high dose therapy (Macquart-Moulin *et al*, 2000; Brandberg *et al*, 2003; Peppercorn *et al*, 2005). Overall QL improves substantially over the first 2 years (Hurny *et al*, 1996). There is some evidence that dose-dense chemotherapy may be more effective than standard doses (Citron *et al*, 2003), but the impact of such therapy on the QL has not been studied.

For women with early-stage breast cancer and a high risk of relapse, the International Breast Cancer Study Group (IBCSG) compared three cycles of adjuvant dose-intensive epirubicin and cyclophosphamide chemotherapy administered with filgrastim and progenitor cell support (DI-EC) with four courses of standard-dose anthracycline-based chemotherapy followed by three courses of classical CMF (SD-CT) in a randomised clinical trial (Basser *et al*, 2006). There was a nonsignificant trend in favour of DI-EC with respect to disease-free survival. The patients were faced with two markedly different treatment regimens, the shorter dose-intensive chemotherapy requiring prolonged or repeated hospitalisation and the standard therapy of more than double duration as outpatient treatment.

Patients reported indicators of QL longitudinally after commencement of chemotherapy. We also evaluated trade-offs by performing a quality-adjusted survival analysis based on utilities derived from patient-reported health status (Hurny *et al*, 1998), including periods while they were receiving adjuvant therapy, the disease-free interval and, where appropriate, after relapse.

## PATIENTS AND METHODS

### The trial

**Figure 1:**
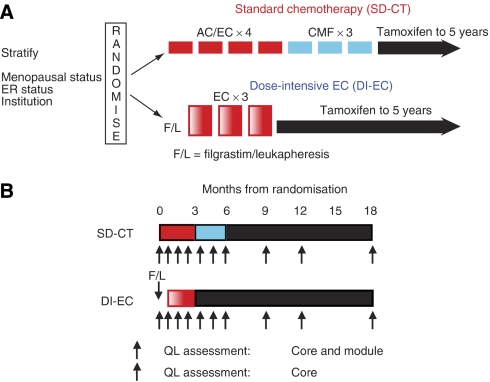
International Breast Cancer Study Group Trial 15–95 schema (**A**). Three hundred forty-four patients were randomised to receive either standard dose chemotherapy (AC or EC × 4 followed by CMF × 3) or dose-intensive EC × 3. All patients were assigned tamoxifen. DI-EC, dose-intensive doxorubicin and cyclophosphamide; SD-CT, standard dose chemotherapy. IBCSG Trial 15–95 QL schema (**B**). Patients were asked to complete a QL core form plus a trial-specific module at baseline, during, and following chemotherapy.

Between July 1995 and March 2000, 344 women with operable breast cancer from centres in Europe, Australia, and Asia were enrolled in IBCSG Trial 15–95 (Figure 1A). Patients were randomised within 6 weeks after surgery and the randomised treatment was to commence within 10 weeks. Standard dose chemotherapy consisted of intravenous injections of doxorubicin 60 mg m^−2^ or epirubicin 90 mg m^−2^ and cyclophosphamide 600 mg m^−2^ (AC or EC) every 3 weeks for four cycles. This was immediately followed by oral cyclophosphamide 100 mg m^−2^ daily for 14 days, and intravenous injections of methotrexate 40 mg m^−2^ and fluorouracil 600 mg m^−2^ on days 1 and 8 (‘classical’ CMF), every 4 weeks for three cycles. In the DI-EC regimen, peripheral blood progenitor cells were collected before chemotherapy. Filgrastim 10 *μ*g kg^−1^ was given subcutaneously daily for 6 days with leukapheresis on days 5, 6, and 7 of administration. Chemotherapy consisted of epirubicin 200 mg m^−2^ intravenously over 1 h on day 1 and cyclophosphamide 4 g m^−2^ on day 2, given as 1 gm m^−2^ intravenously over 30 min in four divided doses. Dose-intensive doxorubicin and cyclophosphamide was given every 3 weeks for three cycles. All patients were assigned to receive tamoxifen 20 mg per day through 5 years once chemotherapy had finished.

Informed consent was obtained from all patients, and the institutional review committee at each participating centre approved the study. The details of the trial protocol and conduct are described elsewhere (Basser *et al*, 2006).

### Quality of life

The protocol required that all patients participate in the QL study. Patients were asked to complete a QL assessment on day 1 of each cycle of chemotherapy. In order to have assessments at approximately the same time points as in the SD-CT group, patients randomised to receive DI-EC were asked to fill in two baseline assessments, one on day 1 of Filgrastim and another on day 1 of the first EC cycle. To assess morbidity associated with treatment, we report QL data for the first 18 months following randomisation in patients without recurrence within this time (Figure 1B).

The original hypothesis (1995) was that patients receiving DI-EC have a worse QL than those receiving SD-CT for at least 24 months. On the basis of later findings (Macquart-Moulin *et al*, 2000; Del Mastro *et al*, 2002; Brandberg *et al*, 2003; Peppercorn *et al*, 2005), we predicted worse QL during DI-EC as compared to SD-CT but a rapid recovery and no treatment-related differences 3 months after completion of chemotherapy. No formal power calculations were made at the time the study was initiated. Coping effort (Hurny *et al*, 1993) had been selected as the primary end point. This global indicator (‘How much effort does it cost you to cope with your illness?’) in the linear analogue self-assessment (LASA) format was selected due to its responsiveness to chemotherapy, changes over time on and off treatment (Hurny *et al*, 1996), and psychological distress (Bernhard *et al*, 2001).

Aspects of QL were assessed by the IBCSG QL core questionnaire (Bernhard *et al*, 1997) comprising single-item LASA indicators for physical well-being, mood, coping effort, appetite, tiredness, hot flushes, nausea/vomiting, perceived social support, restrictions in arm movement, and subjective health estimation (SHE) (Hurny *et al*, 1998). In addition, a trial-specific module for the first 9 months after randomisation included LASA indicators for hair loss, numbness, thought of actually having treatment, loss of sexual interest or ability, all based on GLQ-8 (Coates *et al*, 1990), sore mouth, and pain. Finally, two global LASA indicators were included in the trial-specific module, one for overall treatment burden (Bernhard *et al*, 2002) (‘Overall, how much are you bothered by any treatment related difficulties?’) and an *ad hoc* item of functional performance (‘How well do you perform your daily activities as compared with the time before your breast operation?’). The burden indicator has shown itself to be less precise for specific toxicity effects but responsive to the whole spectrum of treatment sequelae (Bernhard *et al*, 2002).

Completing and submitting a baseline QL assessment before randomisation was not an eligibility requirement. As a result, 87% of the patients with a baseline QL assessment completed the form after knowing their assigned treatment. As a substantial number of patients completed their baseline assessment after randomisation, we did not test for differences in QL scores between baseline and subsequent time points.

A linear mixed-effects model assuming a spatial covariance structure was used to estimate and describe treatment effects of QL over time and at specific time points. This model used all the QL information obtained on the patient while allowing for the repeated measurements to exhibit correlation between observations attributable to within the subject. Missing QL data were assumed to be missing at random. We used the square roots of the QL scores to reduce skewing and to stabilise the variances for all indicators. The figures show the results on the original scale from 0 to 100, with higher scores indicating a better condition. We report and plot the model-based mean estimates and the corresponding 95% confidence intervals of the treatment effects adjusted for patients’ culture (Hurny *et al*, 1996; Bernhard *et al*, 1998), age, and baseline score (see Table 1 for culture definitions). We considered a change of ⩾8 points on a scale of 0–100 as clinically significant (Sloan and Dueck, 2004).

### Quality-adjusted survival

We used the Q-TWiST model, which divides the lifespan of the patients from the beginning of adjuvant treatment until death into three time segments corresponding to distinct health states: TOX (time with toxicity from adjuvant treatment), TWiST (time without reported symptoms of treatment or disease), and REL (time from first relapse until death) (Goldhirsch *et al*, 1989). To calculate Q-TWiST, each health state is assigned a utility coefficient (*u*_t_, *u*_twist_, and *u*_r_), which gives a value to time spent in the state relative to the value of an equal amount of time spent in a state of ‘perfect health.’ The utilities are assumed to be in the interval (0,1), where a zero indicates the worst possible health and one indicates a state as good as perfect health. The Q-TWiST value is the linear combination of the health state durations adjusted by the respective utilities: Q-TWiST=*u*_t_ × TOX+*u*_twist_ × TWiST+*u*_r_ × REL.

We calculated the time spent in the three health states for 324 of the 344 randomised patients, that is, we excluded the 20 patients who never received any of their prescribed chemotherapy (6 in the SD-CT group and 14 in the DI-EC group). Toxicity was assessed and collected during chemotherapy. One month was assigned to TOX for any reversible subjective toxic effect (i.e., does not include objective laboratory measures, e.g., blood count) of grade 3 or higher reported during a cycle. Three additional months were included after the last report of grade 3 or higher alopecia or weight gain. Additional time was added to TOX for subjective grade 3 and higher toxicities reported after chemotherapy ended for 13 cases in the DI-EC group and 1 case in the SD-CT group. Appendix A describes the QL-oriented end points and defines toxicity included in this analysis.

All available SHE scores (Hurny *et al*, 1998) obtained from these 324 patients were used to calculate the utilities attached to each of the three health state durations. For the first 18 months (Figure 1B) and yearly from months 24 to 72, patients were asked to imagine that they would have to live the rest of their life in their current condition and then to rate this condition between ‘perfect health’ and ‘worst health.’ This indicator was previously validated against a time trade-off interview in patients with metastatic breast cancer (Hurny *et al*, 1998). Within each health state, we calculated the median SHE score using all available scores averaged within patients; that is, for TOX the first 6 months on SD-CT and months 2 and 3 on DI-EC. These health estimates were converted to quality weights using a power transformation (Bernhard *et al*, 2004). As a supplemental analysis, SHE scores were averaged separately within those patients reporting any subjective grade 3 or higher toxicity during chemotherapy. To account for the less than ‘perfect health’ during the TWiST state, we divided the utilities of both analyses by the respective *u*_twist_ so that Q-TWiST is interpreted relative to TWiST: Q-TWiST=(*u*_t_/*u*_twist_) × TOX+(*u*_twist_/*u*_twist_) × TWiST+(*u*_r_/*u*_twist_) × REL.

Mean health state durations were estimated from censored survival data (product limit method) up to 72 months from randomisation by computing the areas between the survival curve estimates for the transition times. These durations were adjusted using the patient-derived utilities in order to estimate the mean Q-TWiST for each treatment group. The variability of the patient-derived utilities was not factored into this estimate. However, we performed a threshold utility analysis for the full range of possible utilities for TOX and REL.

*P*-values are two-sided. *P*⩽0.05 were deemed statistically significant and *P*<0.01 are reported as <0.01. No adjustment was made for multiple testing.

## RESULTS

### Quality of life

The definition of the sample for the QL analysis is shown in Table 1. Baseline characteristics were balanced according to treatment arm (Table 2). Twenty-five percent of the patients were less than 40 years old. Mastectomy was the primary surgery in 69% of women while breast-conserving surgery was performed in 31%. Radiotherapy was administered to 49% of the former group and 100% of the latter group. The QL form submission rates at baseline and at 3, 6, 9, 12, and 18 months following randomisation were 78, 82, 78, 84, 82, and 85%, respectively. More patients in the SD-CT group had baseline QL data (89 *vs* 68%); otherwise, the QL form submission rates were similar between treatment arms. Participants and non-participants at month 18 were similar regarding age, menopausal status, tumour size, tumour grade, and ER status at random assignment.

There were no baseline differences between treatments (Table 3). Baseline scores were most impaired for the primary QL end point (coping effort) and also for secondary indicators, including the thought of having treatment.

**Figure 2:**
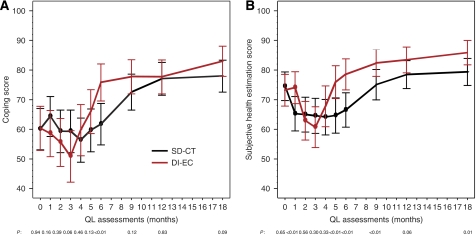
Mean estimates of coping (**A**) and SHE scores (**B**) by treatment group during the first 18 months. Estimates were obtained from a linear mixed-effects model adjusted for patients’ age, culture, and baseline score. The points indicate the assessments when the patients were undergoing chemotherapy. Higher values indicate less effort to cope or better health status (i.e., better condition).

**Figure 3:**
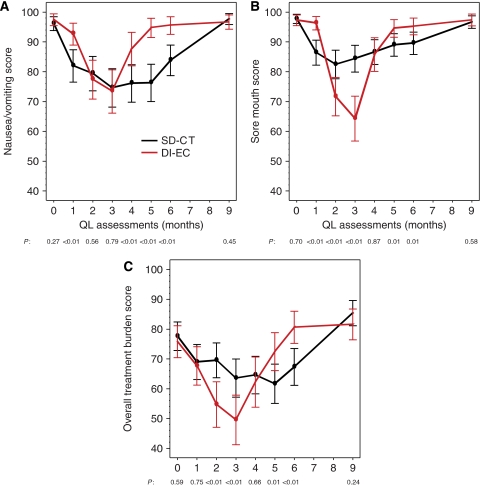
Mean estimates of nausea/vomiting (**A**), sore mouth (**B**), and overall treatment burden scores (**C**) by treatment group during the first 9 months. Estimates were obtained from a linear mixed-effects model adjusted for patients’ age, culture, and baseline score. The points indicate the assessments when the patients were undergoing chemotherapy. Higher values indicate less side effects or burden (i.e., better condition).

In both the SD-CT and DI-EC groups, the global QL indicators showed a noticeable reduction while on treatment but a marked improvement 3 months following chemotherapy to levels generally exceeding those reported at baseline. This pattern across time was more pronounced for DI-EC, as shown for coping effort and SHE (Figure 2A and B, respectively). At the first month on treatment for the SD-CT cohort, but before the DI-EC cohort received protocol therapy, better scores were seen for patients receiving DI-EC as compared with those receiving SD-CT. Substantial differences in patient perception of side effects were present, particularly after completion of the shorter DI-EC therapy, as shown for nausea/vomiting and sore mouth (Figure 3A and B, respectively). Worse scores for sore mouth were evident at months 2 and 3 for the DI-EC cohort and better scores at months 5 and 6 following the cessation of DI-EC treatment but before the end of SD-CT. Patients’ overall estimates of treatment burden (Figure 3C) showed a similar pattern, with worse scores for those with DI-EC at months 2 and 3 (both *P*<0.01) and better scores at months 5 and 6 (*P*=0.01 and <0.01, respectively).

Table 3 shows the estimated mean scores derived from the linear mixed-effects models adjusted for patients’ culture, age, and baseline score at selected time points for all QL indicators. This analysis confirms that time had a major impact on patients’ QL, which differed according to treatment. The time by treatment interaction estimate was statistically significant for all indicators. In particular, the improvement over time following chemotherapy is seen on both arms, but a significantly larger and more rapid recovery is seen within the DI-EC cohort as compared with the SD-CT cohort. For instance, the change in coping scores after completion of therapy on the DI-EC arm improved by 25 U from month 3 to 6 (*P*<0.01), whereas on the SD-CT arm, coping scores improved by 11 U from month 6 to 9 (*P*<0.01). These changes in coping exceeded the minimal clinical significance, with an average improvement of 14 U more in the DI-EC cohort compared with those in the SD-CT cohort (*P*<0.01). There were similar treatment differences in recovery by most of the other indicators (data not shown). At month 9, there was no difference by treatment with the exception of subjective health (*P*=0.01). Patients in the DI-EC cohort reported consistently better health estimates over the whole follow-up (Figure 2B).

### Quality-adjusted survival

Baseline characteristics for the 324 patients included in the Q-TWiST analysis were balanced according to treatment arm and were similar to the baseline characteristics of the 243 patients included in the QL analysis (Table 2). Unadjusted disease-free and overall survival at 72 months median follow-up are summarised in Table 4. Although the result was not statistically significant, patients who received DI-EC had improved disease-free survival compared with patients who received SD-CT (*P*=0.11).

The estimated utility coefficients derived from all available patients for the TOX, TWiST, and REL states were 0.77, 0.91, and 0.77, respectively (Table 5). Fifty-two percent of the patients receiving DI-EC experienced at least one grade 3 or higher subjective toxic side effect during chemotherapy; 18% on SD-CT. For a more conservative estimate, we derived utility scores for TOX from this subsample, which were similar to the total sample, but more pronounced (*u*_t_=0.70; Table 5).

Using the estimates of all available scores (*u*_t_=0.77), the average Q-TWiST for patients receiving DI-EC was 52.1 months, 1.8 months longer than for patients receiving SD-CT (95% CI, −2.5 to 6.1; Table 6). Similarly, using *u*_t_ separately from those patients reporting any subjective grade 3 or higher toxicity (*u*_t_=0.70), the average Q-TWiST for patients receiving DI-EC was 51.9 months, again 1.7 months longer than for SD-CT (95% CI, −2.7 to 6.0).

In further analyses accounting for less than ‘perfect health’ during the TWiST state, the average Q-TWiST for patients receiving DI-EC was 57.1 months, yielding a similar estimate of 2.0 months longer than for patients receiving SD-CT (95% CI, −2.8 to 6.7). The adjusted coefficients from those patients reporting any subjective grade 3 or higher toxicity resulted in an average Q-TWiST for patients receiving DI-EC of 57.1 months, 1.8 months longer than for those receiving SD-CT (95% CI, −2.9 to 6.6).

**Figure 4:**
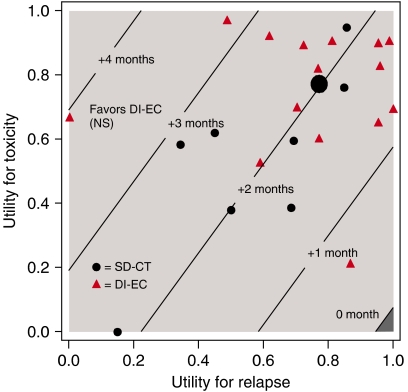
Threshold utility analysis (*N*=324). The utility for TWiST is defined as *u*_TWiST_=1 (i.e., reference state). The solid line labelled zero is the threshold above which DI-EC results in improved Q-TWiST compared with SD-CT. The area below zero (darker area) represents improved Q-TWiST for SD-CT. The larger dot represents the overall estimated patient-rated utilities (*u*_t_=0.77 and *u*_r_=0.77), while the smaller dots (SD-CT) and triangles (DI-EC) illustrate the available individual patient-rated utilities by treatment for those cases that experienced both grade⩾3 toxicity during chemotherapy and disease relapse (*N*=23).

Figure 4 displays the threshold plot comparing DI-EC to SD-CT, which presents treatment comparisons for all possible values of the utilities for TOX and REL, allowing the interpretation of the trial results to be based on individual preferences. In order to visualise the patients’ own perception of their health status (Table 5), the overall estimated patient-rated utilities (*u*_t_=0.77 and *u*_r_=0.77) have been superimposed. In addition, to illustrate the variability around these overall estimates, we superimposed the patient-rated utilities for those cases that experienced both grade 3 or higher toxicity during chemotherapy and disease relapse (*N*=23). The diagonal lines indicate the units of months gained in Q-TWiST for a given pair of TOX and REL utilities. For example, a conventional treatment comparison for disease-free survival is restricted to the TOX and TWiST states, which are valued the same (*u*_t_=*u*_twist_=1). On the basis of these assumptions, there is a statistically nonsignificant gain for DI-EC of less than 2 months. Taking into account the different values for TOX (*u*_t_<1) and TWiST (*u*_twist_=1) and including REL, Q-TWiST would favour DI-EC for almost all values of *u*_t_ and *u*_r_, although the results did not achieve statistical significance.

## DISCUSSION

We have compared QL indicators and quality-adjusted survival in patients receiving a dose-intensive but shorter chemotherapy regimen with those receiving a less toxic standard regimen. The therapies showed distinctly different patterns, reflecting both the delayed commencement and shorter duration of DI-EC. Patients receiving both SD-CT and DI-EC showed rapid recovery in QL to levels generally exceeding those recorded at baseline. Despite the greater initial toxicity of the DI-EC regimen, there were minimal treatment-related differences after completion of chemotherapy, a finding consistent with previous trials (Macquart-Moulin *et al*, 2000; Del Mastro *et al*, 2002; Brandberg *et al*, 2003; Peppercorn *et al*, 2005).

Because we wished to obtain detailed QL scores during treatment, we used monthly QL assessments during chemotherapy and the first 3 months of follow-up. This shorter schedule with more frequent QL assessments revealed a time by treatment interaction sensitive to the duration of treatment: patients receiving DI-EC showed worse QL scores during the time of chemotherapy but a faster recovery following the shorter duration of treatment, while patients receiving SD-CT showed a less pronounced worsening but a more protracted recovery. It is possible that the marked improvement in coping during the initial months after completing DI-EC reflects the relief and sense of accomplishment that the patient may feel after undergoing a difficult experience. Adaptation and thus time itself can have a decisive impact on mental and physical functioning in early breast cancer (Hurny *et al*, 1996; Helgeson *et al*, 2004), which may come into play less under chemotherapy. Informing the patient about this improved outlook is of great value when guiding her through chemotherapy and caring for acute toxicity.

To evaluate trade-offs between QL and survival time, we used utility scores derived from the SHE scale (Hurny *et al*, 1998) during adjuvant therapy, disease-free interval, and after relapse with the time spent in each state and performed a quality-adjusted survival analysis. During the TOX and the TWiST states, the patient-derived utility values were similar between the two regimens. Although the utilities in the TWiST state were better than during TOX, they were less than ‘perfect,’ comparable to those in patients with node-negative disease receiving tamoxifen only or three cycles of CMF (Bernhard *et al*, 2004). In addition to the threat of a malignant disease, menopausal symptoms, fatigue (Ganz *et al*, 2002; Fan *et al*, 2005; Nieboer *et al*, 2005), and cognitive impairment (Phillips and Bernhard, 2003; Falleti *et al*, 2005; Stewart *et al*, 2006), and their secondary effects can impact general well-being in the long run. For nearly all values of weights applied to TOX or REL, because of recovery from the shorter duration DI-EC therapy and its influence on delaying relapse, the threshold utility analysis favoured DI-EC, further supporting the clinical study's overall conclusion (Basser *et al*, 2006).

Although patients were aware of their assigned treatment, there were no baseline differences. Under treatment, our findings are confounded by the setting of care, that is, in-patient for DI-EC *vs* out-patient for SD-CT, as reflected in the finding that patients in the DI-EC group felt more supported during chemotherapy. Overall, the pattern of QL differences between the two regimens was consistent. Our QL follow-up was restricted to 18 months, and thus we could not evaluate the long-term sequelae of the two regimens.

We recommend complementing a QL analysis by patient-weighted Q-TWiST analysis, particularly for phase III trials on regimens with distinct toxicity profiles.

In conclusion, taking into account not only the extent of toxicity during and following chemotherapy but also the duration, patients’ perception of the more intensive DI-EC regimen is more favourable than that of SD-CT. Our findings may be generalised for similar future dose-intensive regimens used in breast cancer and those currently in use for other cancer sites (e.g., lymphomas). The role of shorter total time on chemotherapy with some intense treatment regimens has implications for adaptation but has not received the attention it deserves. In particular, the rapid recovery of QL after a more toxic but shorter regimen needs further study.

## Figures and Tables

**Table 1:** Description of patients excluded from the QL analysis

	**Remaining cases**	
Total cases		344
Exclusions from analysis:		
Did not receive protocol treatment	20	324
Relapse within first 18 months of randomisation	72	252
Completed no QL assessments	3	249
Completed assessments in multiple languages	6	243

Abbreviations: QL=quality of life.

Culture is defined by the following language/country combinations: English/Australia, New Zealand; French/Switzerland; German; Italian/Italy; Slovenian/Slovenia; Hong Kong.

**Table 2:** Baseline characteristics of the 243 patients included in the QL analysis and of the 324 patients included in the Q-TWiST-analysis

	**243 Patients included in the QL analysis**						**324 Patients included in the QAS analysis**					
	**DI-EC**		**SD-CT**		**Total**		**DI-EC**		**SD-CT**		**Total**	
	***N***	**(%)**	***N***	**(%)**	***N***	**(%)**	***N***	**(%)**	***N***	**(%)**	***N***	**(%)**
Total cases	121	(100)	122	(100)	243	(100)	159	(100)	165	(100)	324	(100)
ER-negative	54	(45)	61	(50)	115	(47)	83	(52)	92	(56)	175	(54)
ER-positive	67	(55)	59	(48)	126	(52)	76	(48)	71	(43)	147	(45)
ER-unknown	0	(0)	2	(2)	2	(1)	0	(0)	2	(1)	2	(1)
												
Age <40 years	32	(26)	28	(23)	60	(25)	41	(26)	36	(22)	77	(24)
Age ⩾40 years	89	(74)	94	(77)	183	(75)	118	(74)	129	(78)	247	(76)
												
Total mastectomy no. of RT	48	(40)	38	(31)	86	(35)	64	(40)	53	(32)	117	(36)
Total mastectomy+RT	41	(34)	42	(34)	83	(34)	55	(35)	55	(33)	110	(34)
Breast conservation no. of RT	0	(0)	0	(0)	0	(0)	0	(0)	1	(1)	1	(<1)
Breast conservation+RT	32	(26)	42	(34)	74	(31)	40	(25)	56	(34)	96	(30)
												
Premenopausal	81	(67)	84	(69)	165	(68)	107	(67)	111	(67)	218	(67)
Postmenopausal	40	(33)	38	(31)	78	(32)	52	(33)	54	(33)	106	(33)

Abbreviations: DI-EC=dose-intensive epirubicin cyclophosphamide; QL=quality of life; RT=radiotherapy; SD-CT=standard dose chemotherapy (AC × 4-CMF × 3).

**Table 3:** Mean estimates of each QL indicator from the (a) IBCSG Core Form and (b) trial-specific module by treatment group at months 0, 3, 6, 9, 12, and 18 months from randomisation^a^

	**Months from randomisation**																	
	**0**			**3**			**6**			**9**			**12**			**18**		
**QL**	**SD-CT**	**DI-EC**	***P*-value**	**SD-CT**	**DI-EC**	***P*-value**	**SD-CT**	**DI-EC**	***P*-value**	**SD-CT**	**DI-EC**	***P*-value**	**SD-CT**	**DI-EC**	***P*-value**	**SD-CT**	**DI-EC**	***P*-value**
(a)																		
Physical well-being	84±2	87±2	0.24	73±3	52±4	<0.01	75±3	84±3	0.01	88±2	87±2	0.76	89±2	89±2	0.90	88±2	90±2	0.30
Mood	74±3	74±3	0.95	74±3	64±4	0.01	76±3	84±3	0.02	85±2	83±2	0.51	86±2	87±2	0.71	88±2	89±2	0.52
Tiredness	76±3	78±3	0.42	56±4	46±5	0.05	59±4	73±4	<0.01	69±3	75±3	0.12	76±3	76±3	0.94	74±3	81±3	0.03
Appetite	89±2	90±2	0.60	81±3	58±4	<0.01	85±2	88±2	0.27	92±2	92±2	0.84	94±2	93±2	0.97	94±1	94±2	0.94
Hot flushes	93±2	94±2	0.65	84±3	90±3	0.05	76±4	79±4	0.60	68±4	71±4	0.44	68±4	77±4	0.05	63±5	70±5	0.16
Nausea/vomiting	96±1	98±1	0.23	74±3	74±3	0.83	84±2	96±1	<0.01	98±1	97±1	0.46	97±1	98±1	0.56	98±1	98±1	0.71
Coping effort	60±4	61±4	0.94	60±4	51±4	0.06	62±4	76±3	<0.01	73±3	78±3	0.12	77±3	78±3	0.83	78±3	83±3	0.09
Feel supported	97±1	97±1	0.87	93±1	96±1	0.04	93±1	96±1	0.07	94±1	94±1	0.91	93±1	94±1	0.40	93±1	95±1	0.18
Arm restriction	71±3	71±3	0.84	83±2	81±3	0.49	85±2	82±3	0.44	83±2	81±3	0.49	80±3	82±3	0.51	84±2	83±3	0.67
Subjective health	75±2	73±3	0.65	65±3	61±4	0.30	67±3	79±3	<0.01	75±3	83±2	0.01	79±2	84±2	0.06	80±2	86±2	0.01
																		
(b)																		
Hair loss	94±2	96±1	0.24	36±5	34±6	0.79	83±3	85±3	0.63	94±2	93±2	0.63						
Numbness	92±2	93±1	0.63	88±2	90±2	0.43	89±2	88±2	0.81	91±2	87±2	0.16						
Thoughts	60±3	58±4	0.66	63±3	53±4	0.05	70±3	84±3	<0.01	89±2	89±2	0.83						
Sexual interest	81±3	77±3	0.38	64±4	62±4	0.76	63±4	80±3	<0.01	79±3	83±3	0.28						
Sore mouth	98±1	98±1	0.70	85±2	65±4	<0.01	90±2	96±1	0.01	97±1	98±1	0.58						
Pain	90±2	91±2	0.74	88±2	84±2	0.11	88±2	91±2	0.34	90±2	92±2	0.44						
Treatment burden	78±2	76±3	0.59	64±3	50±4	<0.01	68±3	81±3	<0.01	86±2	82±3	0.24						
Daily activity	74±2	74±3	0.99	68±3	52±4	<0.01	73±3	79±3	0.06	82±2	84±2	0.49						

Abbreviations: QL=quality of life; DI-EC=dose-intensive epirubicin cyclophosphamide; SD-CT=standard dose chemotherapy (ACx4-CMFx3). Higher scores indicate a better condition for all indicators.

a Estimates were obtained from a linear mixed-effects model adjusted for patients’ age, culture, and baseline score.

**Table 4:** Unadjusted disease-free and overall survival by treatment at 72 months median follow-up for the 324^a^ patients included in the Q-TWiST-analysis

	**No. of patients**	**No. of events**	**5-year DFS%±s.e.**	***P*-value**	**No. of dead**	**5-year OS%±s.e.**	***P*-value**
DI-EC	159	79	52±4	0.11	55	71±4	0.25
SD-CT	165	97	44±4		67	63±4	

Abbreviations: DI-EC=dose-intensive epirubicin cyclophosphamide; SD-CT=standard dose chemotherapy (ACx4-CMFx3); DFS=disease-free survival; OS=overall survival; s.e.=standard error; *P*=*P*-value.

a Excluding 20 patients who did not receive any of their prescribed treatment (6 in the SD-CT group and 14 in the DI-EC group).

**Table 5:** Patient-derived utility coefficients: overall and according to treatment

**Health state**	***N* a**	**SHE**	**TTO=1−(1−SHE)^1.6^**
Mean SHE scores within patients with SD-CT (months: 1, 2, 3, 4, 5, 6) or DI-EC (months 2, 3)			
*TOX*			
Total sample	284	0.60	0.77
SD-CT	149	0.60	0.77
DI-EC	135	0.57	0.74
			
Mean SHE scores within patients reporting grade 3 or higher toxicity with SD-CT (months: 1, 2, 3, 4, 5, 6) or DI-EC (months 2, 3)			
*TOX*			
Total sample	96	0.53	0.70
SD-CT	27	0.51	0.68
DI-EC	69	0.55	0.72
			
*TWiST*			
Total sample	292	0.78	0.91
SD-CT	140	0.80	0.92
DI-EC	152	0.77	0.90
			
*REL*			
Total sample	85	0.60	0.77
SD-CT	51	0.55	0.72
DI-EC	34	0.64	0.80

Abbreviations: DI-EC=dose-intensive epirubicin cyclophosphamide; SD-CT=standard dose chemotherapy (ACx4-CMFx3); SHE=subjective health evaluation; TTO=time trade-off.

a Sample size reflects those patients who experienced that health state and who responded to the SHE question at least once during that health state.

**Table 6:** Averaged months of TOX, TWiST, and REL accumulated within 72 months of randomisation, with Q-TWiST calculated for patient-derived utility coefficients (*N*=324^a^)

	**Treatment group**			
	**DI-EC**	**SD-CT**		
	**Average months**	**Average months**	**Difference**	**95% CI**
*Components of Q-TWiST*				
TOX^b^	2.32 (0.57)	0.32 (0.06)	—	—
TWiST	47.25 (2.11)	44.55 (2.07)	—	—
REL	9.44 (1.25)	12.28 (1.25)	—	—
				
*Q-TWiST=u*_*t*_ × *TOX+u*_*twist*_ × *TWiST+u*_*r*_ × *REL*				
All available scores: (*u*_t_=0.77, *u*_twist_=0.91, *u*_r_=0.77)	52.05 (1.55)	50.24 (1.53)	1.81 (2.21)	(−2.51–6.14)
Grade 3 or higher toxicity: (*u*_t_=0.70, *u*_twist_=0.91, *u*_r_=0.77)	51.89 (1.55)	50.22 (1.53)	1.67 (2.21)	(−2.66–6.00)
All available scores, adjusted for ‘less than perfect’: (*u*_t_=0.85, *u*_twist_=1.00, *u*_r_=0.85)	57.25 (1.71)	55.26 (1.68)	1.99 (2.43)	(−2.77–6.74)
Grade 3 or higher toxicity, adjusted for ‘less than perfect’: (*u*_t_=0.77, *u*_twist_=1.00, *u*_r_=0.85)	57.06 (1.71)	55.23 (1.68)	1.83 (2.43)	(−2.93–6.58)

Abbreviations: CI=confidence interval; DI-EC=dose-intensive epirubicin cyclophosphamide; SD-CT=standard dose chemotherapy (ACx4-CMFx3).

Note: standard errors are shown in parentheses. Standard errors do not factor in the variability of the patient-derived utility coefficients.

a Excluding 20 patients who did not receive any of their prescribed treatment (6 in the SD-CT group and 14 in the DI-EC group).

b One month was assigned to TOX for any reversible subjective toxic effect (i.e., does not include objective laboratory measures, e.g., blood counts) of grade 3 reported during a cycle. Three additional months were included after last report of grade 3 or higher alopecia or weight gain. For 13 DI-EC cases and 1 SD-CT case that experienced subjective toxicities of grade 3 or higher following chemotherapy, TOX includes the additional months attributable to these late toxicities. See Appendix A for a complete definition of subjective toxicity.

**Table A1:** 

**Event**	**Abbreviation**	**Time assigned to**
Any reversible subjective toxic effect^a^ of grade 3 or higher during chemotherapy	TOX	Applied to the entire month
Alopecia and weight gain during chemotherapy	TOX	Include an additional 3 months after last report to allow for recovery
Any subjective toxic effect^a^ of grade 3 or higher following chemotherapy	TOX	Include the total duration of toxic effect until resolution
Any relapse (including ipsilateral breast relapse) or the appearance of a second primary cancer	REL	Applies to entire remaining survival period
None of the above	TWiST	Applies to any remaining survival time

a Subjective toxic effects include those noted and graded by the investigators: nausea, vomiting, diarrhoea, stomatitis, mucositis, haemorrhage, vaginal bleeding, infections, anorexia, epigastric pain, pulmonary, neurotoxicity, depression, skin allergy, alopecia, cystitis, headache, muscle weakness, hypercalcaemia, hypertension, hot flashes, euphoria, depression, thrombosis, phlebitis, embolism, oedema, lymphedema, weight gain, eye disorders, joint pain, wound healing, bone pain, fever, cardiac rhythm, cardiac function, pericarditis, cardiac failure, colitis, hypothyrosis, dental damage, post-RT pneumonitis, peripheral neuropathy, fatigue.

**Table B1:** 

Study Chair	R Basser
Scientific Committee	A Goldhirsch, AS Coates (Co-Chairs)
Foundation Council	B Thürlimann (President), M Castiglione-Gertsch, AS Coates, JP Collins, H Cortés Funes, RD Gelber, A Goldhirsch, M Green, A Hiltbrunner, SB Holmberg, DK Hossfeld, P Karlsson, I Láng, J Lindtner, M de Stoppani, C-M Rudenstam, R Stahel, H-J Senn, A Veronesi
Coordinating Center (Bern, Switzerland)	M Castiglione-Gertsch (CEO), A Hiltbrunner (Director), G Egli, M Rabaglio, R Studer, B Ruepp, M Schärlig-Strausak, R Maibach
Statistical Center (Harvard School of Public Health and Dana-Farber Cancer Institute, Boston, MA, USA)	RD Gelber (Group Statistician), K Price (Director of Scientific Administration), A O’Neill (Study Statistician), S Gelber, D Zahrieh, M Bonetti, A Keshaviah, M Regan
Quality of Life Office (Bern, Switzerland)	J Bernhard, Ch. Hürny, H Gusset, G Egli
Pathology Office	B Gusterson, G Viale, V Spataro
Data Management Center (Frontier Science & Tech. Res. Found., Amherst, NY, USA):	L Blacher (Director), J Celano, M Isley, R Hinkle, S Lippert, K Scott
**Australian New Zealand Breast Cancer Trials Group (ANZ BCTG)**	JF Forbes, AS Coates, A Wilson, D Lindsay, D Preece
-Statistical Center, NHMRC CTC, University of Sydney	RJ Simes, V Gebski, H Dhillon
-The Cancer Council Victoria, Melbourne, VIC	J Collins, R Snyder, E Abdi, R Basser, WI Burns, M Chipman, J Chirgwin, V Ganju, M Green, S McLachlan, D Howell, M Prince, A Schwarer, G Toner, C Underhill
-Canberra Hospital, Canberra, ACT	P Craft, S Harris, R Pembrey
-Newcastle Mater Misericordiae Hospital, Waratah, Newcastle, NSW	JF Forbes, J Stewart, S Ackland, A Bonaventura
-Mater Adult Hospital, Brisbane, QLD	K Taylor
-Prince of Wales Hospital, Randwick, NSW	M Friedlander, B Brigham, C Lewis, D Goldstein
-Queen Elizabeth Hospital, Woodville, SA	D Kotasek
-Royal Adelaide Hospital, Adelaide, SA,	IN Olver, PG Gill, D Keefe
-Royal Brisbane Hospital, Brisbane, QLD	R Abraham, D Wyld
-Royal North Shore Hospital, St Leonards, NSW	F Boyle, S Durrant, D Bell
-Royal Prince Alfred Hospital, Sydney	J Beith, M Boyer, AS Coates, A Sullivan
-Sir Charles Gairdner Hospital, Nedlands, WA	M Byrne, G van Hazel, J Dewar
-Auckland Hospital, Auckland, New Zealand	VJ Harvey, P Thompson, D Porter, M McCrystal
**Istituto Europeo di Oncologia, Milano, Italy**	G Martinelli, F Peccatori, U Veronesi, G Viale, A Luini, R Orecchia, S Cinieri, E Cocorocchio, G Renne, G Mazzarol, M Colleoni, A Agazzi, F Nolè, A Costa, S Zurrida, P Veronesi, V Sacchini, V Galimberti, F de Braud, G Peruzzotti, F Didier, A Goldhirsch
**Ospedale Infermi, Rimini, Italy**	A Ravaioli, D Tassinari, G Oliverio, F Barbanti, P Rinaldi, L Gianni, G Drudi
**The Chinese University of Hong Kong**	N Wickham, T Leung, W Yeo, W King, W Kwan, M Suen, K Chak, L Lee
**SAKK (Swiss Group for Clinical Cancer Research)**	
-Inselspital, Bern	MF Fey, E Dreher, H Schneider, S Aebi, K Buser, J Ludin, G Beck, H Bürgi, A Haenel, JM Lüthi, R Markwalder, HJ Altermatt, M Nandedkar
-Kantonsspital, St Gallen	HJ Senn, B Thürlimann, Ch. Oehlschlegel, G Ries, M Töpfer, U Lorenz, B Späti
-IOSI (Istituto Oncologico della Svizzera Itraliana) Ospedale San Giovanni, Bellinzona	F Cavalli, C Sessa, G Martinelli, M Ghielmini, P Luscieti, J Bernier, E Pedrinis, T Rusca; A Goldhirsch
-Centre Hôpitalier Universitaire, Lausanne	L Perey, S Leyvraz, P Anani, C Genton, F Gomez, P De Grandi, P Reymond, R Mirimanoff, M Gillet, JF Delaloye
-Hôpital Cantonal, Geneva	P Alberto, H Bonnefoi, P Schäfer, F Krauer, M Forni, M Aapro, R Egeli, R Megevand, E Jacot-des-Combes, A Schindler, B Borisch, S Diebold
-Kantonsspital, Zürich	Ch. Sauter, U Metzger, V Engeler, U Haller, R Caduff
**The Institute of Oncology, Ljubljana, Slovenia**	J Lindtner, D Eržen, E Majdic, B Stabuc, A Plesnicar, R Golouh, J Lamovec, J Jancar, I Vrhoved, M Kramberger

## Appendix A

### DEFINITION OF QUALITY-OF-LIFE ORIENTED END POINTS

Table A1

## Appendix B

### PARTICIPANTS AND AUTHORS TRIAL 15–95

Table B1
